# Folding and assembly of large macromolecular complexes monitored by hydrogen-deuterium exchange and mass spectrometry

**DOI:** 10.1186/1475-2859-7-12

**Published:** 2008-04-04

**Authors:** Bohumila Suchanova, Roman Tuma

**Affiliations:** 1 Institute of Biotechnology, University of Helsinki, Finland; 2 Astbury Centre for Structural Molecular Biology, University of Leeds, UK

## Abstract

Recent advances in protein mass spectrometry (MS) have enabled determinations of hydrogen deuterium exchange (HDX) in large macromolecular complexes. HDX-MS became a valuable tool to follow protein folding, assembly and aggregation. The methodology has a wide range of applications in biotechnology ranging from quality control for over-expressed proteins and their complexes to screening of potential ligands and inhibitors. This review provides an introduction to protein folding and assembly followed by the principles of HDX and MS detection, and concludes with selected examples of applications that might be of interest to the biotechnology community.

## Introduction

Many essential cellular activities are mediated by large protein complexes. Similarly, viruses, the cellular parasites that are often detrimental to human and animal health, constitute a special but important class of large macromolecular assemblies. Such assemblies are composed of multiple protein subunits and may also contain nucleic acids (e.g. ribosome, viruses, chromatin) and lipids (certain viruses and membrane complexes like nuclear pore, receptors, respiratory complexes). As with any protein successful folding of the subunits and assembly into the final complex are the pre-requisites for attaining activity while non-specific aggregation interferes with function and often triggers a cascade of secondary responses that are detrimental to cells and organisms (e.g. apoptosis, neurodegeneration). In certain cases aggregation produces highly structured and stable assemblies (e.g. amyloid fibrils) that in effect represent an alternative outcome of folding and assembly under the altered conditions [1,2]. In all cases, subunit-subunit interactions determine the outcome of assembly and stability of the final complexes. Given the complexity of large assemblies new approaches to investigate structure and dynamics are needed.

Hydrogen deuterium exchange (HDX), which probes accessibility and local dynamics of polypeptide chains, is a powerful method to study protein folding. With the advent of mass spectrometric (MS) detection the method (further referred to as HDX-MS) became amenable to study assembly and structure of large macromolecular complexes. In this contribution, we first provide basic concepts of protein folding and assembly that are necessary for understanding HDX principles. Then we explain the theoretical basis and practical methods for HDX-MS. In the later part we illustrate how HDX-MS can be utilized for characterizing protein folding, misfolding, aggregation, and for probing interactions in large macromolecular complexes.

### Protein folding and assembly

The process of macromolecular assembly starts when the subunit polypeptide chain is made on the ribosome and folds. During early stages of folding the number of accessible conformations is enormous. Experimental studies demonstrated that a rapid collapse of hydrophobic protein core and concomitant formation of secondary structure constitute the early events during folding [3-6]. Detailed tertiary (specific side chain contacts and orientations) and quaternary (subunit interfaces) structures usually develop later. These contacts further stabilize the core and exclude solvent (H_2_O) from the protein interior making the hydrophobic core the most stable part of the structure. The formation of the core has been demonstrated by an early protection of the amide protons (NH) against isotope exchange (hydrogen deuterium exchange, HDX, see below for the detailed mechanism) during the folding process. The core also hosts the most stably hydrogen bonded and thus protected peptide groups which exhibit extremely slow amide exchange under equilibrium conditions [7-9]. This is because amide exchange rates are related to the local and global stability [10].

**Figure 1:**
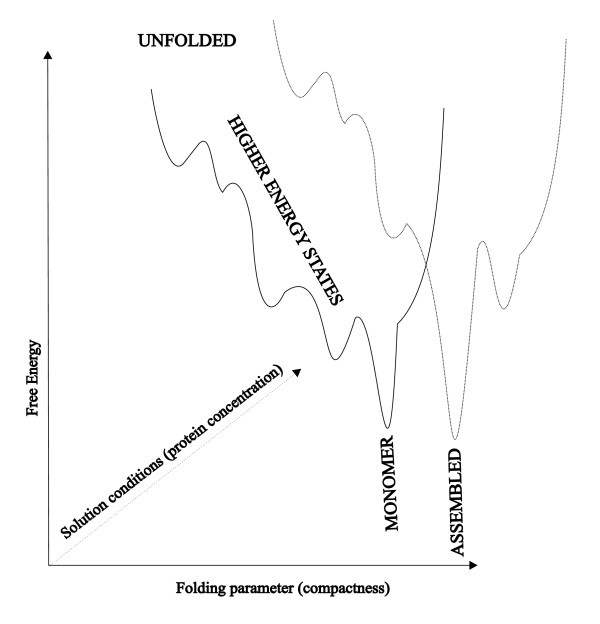
A schematic, one dimensional cross-section of the multidimensional energy landscape projected along a specific folding reaction coordinate.

Protein folding can be best explained with the help of multidimensional energy landscape [11,12]. The folding process is described as successive decrease of polypeptide energy to a global minimum on a landscape which, for a typical globular protein, has a funnel-like shape (Fig. 1 shows a one dimensional cross-section projected along a specific folding parameter such as compactness or percentage of native structure) [13,14]. In vitro experiments and theoretical considerations demonstrated that most protein molecules in the ensemble rapidly adopt their lowest energy state following energetically preferred paths while minority is being slowed down by trapping in local minima on the landscape [15,16]. These kinetically trapped molecules are prone to aggregation [17-19]. Evolutionary pressure selects protein sequences against trapping in local minima and consequently most naturally occurring polypeptides exhibit relatively smooth folding landscapes. However, genetic engineering and over-expression of proteins in heterologous hosts might induce additional roughness into landscapes [20]. This has been manifested by numerous expression studies in which folding behavior was derailed by as little as a single point mutation or by a slight change in folding conditions or absence of correct post-translational modifications [21-27].

Many self-assembling and interacting proteins, however, attain their native fold only upon incorporating into the macromolecular complex [28-30]. The energy landscape for folding and association of self-assembling (or aggregating) proteins depends strongly on protein concentrations and ambient conditions (Fig. 1) [29,31-35]. This is because assembly is often cooperative and relies on multiple weak interactions between many subunits. Weak interactions are sensitive to changes in environmental conditions like temperature, ionic strength and composition and pH. Since amide protection is related to local protein stability [36] HDX constitutes excellent tool for mapping the assembly energy landscape. For example comparison of HDX for subunits in disassembled and assembled states yields appraisal of stabilization by subunit contacts and provides basis for mapping of subunit interfaces.

### Structural characterization of macromolecular complexes

High resolution structure determination relies on experimental techniques such as X-ray crystallography and NMR spectroscopy both of which require substantial protein quantities. The proteins and their complexes are usually produced by expression in a suitable host cell. The most widely used expression hosts are various laboratory strains of *Escherichia coli*, yeast cells or insect cells infected with bacullovirus. Folding of eukaryotic proteins in *E. coli *is often compromised due to the lack of the adequate glycosylation apparatus and appropriate post-translational processing. In such cases one has to resort to expression in cells of higher eukaryots which may significantly decrease the yield to levels insufficient for crystallization or NMR experiments and consequently lower resolution structural techniques, such as electron microscopy (EM), must be used. HDX is an emerging method belonging to this class. In addition, the stoichiometry and structure of the expressed complexes may differ from those occurring in the original cells and tissues. Hence there is a need for methods which allow comparing structure of the expressed complexes with the native, tissue-derived species. HDX and mass spectrometry are well positioned to fill this gap [37,38].

Electron cryo-microscopy offers a suitable alternative for the structure determination of larger complexes (>1 MDa) [39]. However, EM alone seldom provides high enough resolution (i.e. better than 4Å) that would allow building structural models in atomic details. Cryo-EM routinely achieves resolution as high as 7 Å but the resulting electron densities do not allow polypeptide chain tracing and the primary structure (sequence) cannot be mapped onto the structure. Consequently, this provides little information about the residues at subunit interfaces or about the subunit fold. This problem can be circumvented if high resolution structures of the subunits are known and could be fitted into the EM density [40-42]. However, in cases where the high-resolution structures are not available or substantial portions of the structure are not resolved in the models (e.g. intrinsically unfolded domains which become folded upon assembly) one would like to obtain some information about the disposition of amino acids between folding core, subunit interfaces and intrinsically unfolded regions. As indicated above and discussed in more detail in the next section HDX can provide such information. HDX and mass spectrometry (MS) can also help to tackle sample heterogeneity and classification of EM images [43] and thus provides supportive tool to EM [44].

## Theory and Methods

### Principles of hydrogen-deuterium exchange

#### Physical chemistry of amide exchange

**Figure 2:**
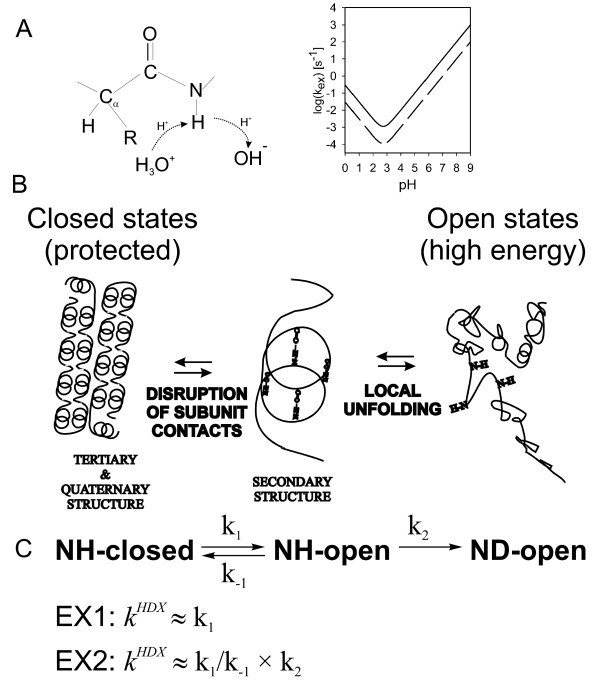
**Mechanism of HDX.** (A) Mechanism of the chemical step. Inset on the right illustrates the pH and temperature (solid line 25°C, dashed line 0°C) dependence of the intrinsic exchange rate (B) Influence of quaternary, tertiary and secondary structure on HDX. (C) Overall reaction scheme and the two limiting cases.

HDX probes the exchange kinetics of the main-chain amide (NH) hydrogens for deuterium in samples exposed to D_2_O (Fig. 2A). The advantage is that, in principle, it provides site-specific probes along the whole polypeptide chain (except prolines and the N-terminal amino group). Under extreme pH conditions (pH<1 or pH>14) a free amide group can be either protonated or ionized [45]. Amide hydrogens can exchange via those states albeit slowly under physiologically relevant conditions. The reaction is greatly accelerated by an acid or a base. In aqueous solutions the hydronium ion (H_3_O^+^) or the hydroxide anion (OH^-^) plays the role of the acid or the base, respectively [45]. The acid- or base-catalyzed substitution of the unprotected (free) amide hydrogen, i.e. the chemical step of the exchange reaction, is strongly pH- and temperature-dependent (Fig. 2A right panel). This means that an exposed amide group would exchange on millisecond time scale at room temperature and neutral pH. Conversely, it would take about an hour to exchange the same exposed NH at low pH = 2.5 and 0°C [46]. The strong pH dependence of the chemical step allows preserving the pattern of labeling that was attained under native, neutral pH conditions by shifting the sample to pH 2.5 and low temperature. This, in turn provides a window of opportunity for the analysis of isotope incorporation by mass spectrometry [46].

Steric hindrance by the neighboring side chains (R in Fig. 2A) also affects the rate of the chemical step. This dependence was calibrated using short, unfolded peptides as model compounds and constitutes relatively small correction to the intrinsic exchange rates of unprotected peptide groups (further designated as k_2 _rate) [47-49]. This correction becomes important when quantitative interpretation for site-specific rates is being sought e.g. determination of the energetics for individual peptide bonds during folding.

#### Effects of protein secondary and tertiary structures

In folded proteins most of the amide hydrogens participate in hydrogen bonding within secondary structure elements, e.g. α-helices and β-sheets (Fig. 2B). This considerably slows down the exchange since it requires temporal breaking of hydrogen bonds. In the folded state secondary structure is further stabilized by tertiary contacts and thus exchange requires partial or complete unfolding of the protein and exposure of the buried segments to solvent. This process is sometimes called structural opening and is a prerequisite for the chemical step of exchange [45]. The open states in Fig. 2B correspond to higher-energy, partially unfolded intermediates on the energy landscape in Fig. 1. Quaternary, subunit contacts provide additional stabilization and protect NH sites on or close to the subunit interfaces [50,51]. In a typical well-folded protein the exchange rates may differ by many orders of magnitude reflecting local and global stability [46].

#### Two step model of amide exchange

The exchange rates are governed by the folding energy landscape. However, the correspondence is not straightforward and it is necessary to consider all factors contributing to the overall exchange rate. In order to simplify such analysis several reasonable assumptions and approximations are made. For amides within protected regions (secondary structure, hydrophobic core, subunit interfaces) structural opening is the rate-limiting step and the exchange process may be considered as a two step reaction (Fig. 2C): (1) structural opening which is governed by rate constants k_1 _and k_-1_. (2) chemical exchange step which is governed by rate k_2 _and considered irreversible under the condition of excess D_2_O. Then the overall HDX rate can be described by:

k_ex _= k_1_k_2_/(k_2_+k_-1_)

There are two limiting cases that are considered when interpreting HDX exchange rates:

(EX1) k_2 _>>k_-1_, i.e. the chemical exchange is fast compared to the structural opening step:

k_ex _= k_1_

The EX1 limit is typical for exchange via global unfolding under strongly denaturing conditions or for subunit dissociation in large assemblies. The rate does not depend on pH. The temperature dependence of the rate yields the activation energy for the unfolding event, i.e. provides information about the heights of barriers (roughness) on the energy landscape.

(EX2) k_-1 _>> k_2_, i.e. the open state is short lived compared to the rate of the chemical step:

k_ex _= k_1_k_2_/k_-1 _= K_op_k_2_

where K_op _is the apparent equilibrium constant for structural opening. EX2 is typical for exchange from the folded state under native or mildly denaturing conditions and is strongly pH dependent (through k_2_). EX2 is a product of the local unfolding equilibrium constant and the chemical step rate constant. When site-specific exchange rates in unfolded state are available it is possible to factor them out and to obtain the protection factor:

p = k_2_/k_ex_

This factor is then related to the local stability, i.e. depths of "wells" on the energy landscape:

ΔG_local _= -*R*·*T*·ln(p)

where *R *is the universal gas constant and *T *is absolute temperature.

Because different mechanisms of local opening usually operate simultaneously, neighboring amides within the same element of secondary structure may exchange with different rates under native conditions. Upon destabilization under progressively more denaturing conditions, these rates converge to a value that is characteristic of the cooperative unfolding unit to which these sites belong [36,52]. This approach identifies structural characteristics of partially unfolded states and enables to map the energy landscape in structural terms [10].

### HDX detection by mass spectrometry

We first compare various methods for HDX detection and then explain practical issues involved in HDX-MS measurements. More detailed discussion of experimental implementation has been provided in recent reviews [46,53,54].

#### Comparison of different methods for HDX detection

Several experimental techniques for measurement of HDX are available. NMR, which determines HDX through disappearance/appearance of amide proton resonances in D_2_O/H_2_O solutions, is the most widely used and provides site-specific probe of protection. Presently, NMR is not applicable to large protein complexes (>200 kD) and requires high concentrations (>10 mg/ml). HDX dynamics of large macromolecular complexes can be readily examined by Fourier transform infrared (FTIR) [55-60] and Raman spectroscopy [29,32,61,62]. These techniques probe incorporation of deuterium by monitoring shifts of frequencies arising from collective vibrations of the peptide bond and the amide NH group (so called amide bands, deuterium incorporation increases the local mass and hence these bands shift to lower frequencies). These techniques differentiate the exchange rates only by the type of secondary structure and do not provide mapping onto the protein sequence.

Mass spectrometry measures deuterium incorporation as an increase of apparent mass, more specifically as a change in the isotopic composition. Advances in protein MS enabled routine resolution of isotopic composition for oligopeptides with masses up to several kDa. Similarly, shifts due to deuterium incorporation are readily discernible in mass spectra of intact proteins. This in turn allowed to probe HDX kinetics for large proteins [63,64] and macromolecular complexes [33,65-68] and map subunit-subunit interactions in assemblies [51,69-72]. Clear advantages of MS detection over NMR are the lower protein concentrations and sample quantities needed. Although, in principle, MS allows for residue-specific resolution of HDX kinetics this is seldom achieved and only region-specific information is obtained [53,73,74].

#### Data acquisition and instrumentation

**Figure 3:**
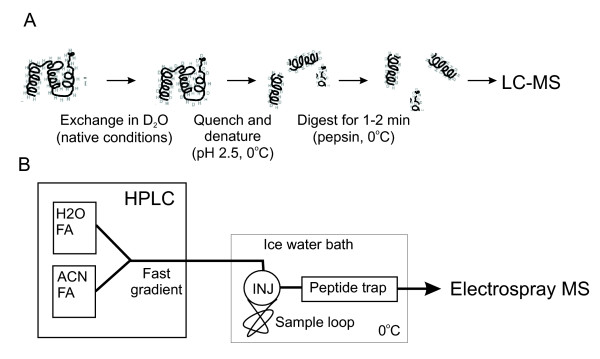
**Practical aspects of HDX measurement. **(A) Simplified exchange and protease digestion protocol. (B) Cooled LC-MS setup, INJ = injector [64].

The extent and rate of HDX is measured from mass increases of peptide fragments after enzymatic cleavage of a protein (Fig. 3). The exchange is usually initiated by diluting the protein into D_2_O exchange buffer (Fig. 3A). The sample is incubated under the exchange conditions for the desired exchange period (usually 30 s to 10 h) and then quenched by rapid acidification to pH 2.5 on ice (usually done with formic acid). This effectively slows down any further exchange (so called exchange-in) and minimizes pickup of hydrogens during subsequent handling in H_2_O solutions (so called back-exchange). The quenching solution may be supplemented with a denaturant (e.g. guanidine hydrochloride or urea) to facilitate dissociation of stable complexes prior to proteolytic digestion. The quenched sample may be analyzed immediately or flash-frozen and stored for up to several weeks in liquid nitrogen for off-line analysis.

Prior to MS analysis the sample is digested with a non-specific acid protease (e.g. pepsin, Aspergillus type XIII, Rhizhopus type XVIII, or their mixture [75]) on ice. The resulting digest is loaded onto a reverse-phase column (usually short C-8 or peptide trap) and rapidly separated by a steep gradient. As shown in Fig. 3B it is essential that the HPLC column and the injector are cooled and kept at constant temperature (e.g. in an ice-water bath) to minimize the back-exchange and maintain reproducible conditions [76]. With the advent of high-resolution MS, good HPLC separation is not essential. The chromatography step effectively provides rapid desalting and removal of undigested protein before sample introduction into the mass spectrometer. This is usually done through a standard or micro-capillary electrospray ionization interface (ESI) [77,78]. Any mass spectrometer with resolving power 5000 or better (e.g. a quadrupole-time of flight (Q-TOF), an ion trap) is in principle suitable for detecting HDX. Higher resolution enables a more accurate measurement of multiply charged peptides and is essential for resolving overlapping isotopic envelopes that are commonly found in MS spectra of large proteins and their assemblies. High mass accuracy and reproducibility, usually provided by calibration with a suitable standard, are essential. The highly repetitive nature of HDX-MS experiment (i.e. collection of many time points) makes it suitable for automation and several setups were reported [53,79].

Note that the exchangeable side-chain hydrogens (e.g. tyrosine or serine OH groups, the amino group of lysine etc.) exchange rapidly back during digestion and HPLC separation performed in H_2_O [80]. On the other hand, deuterium at main-chain amide sites persists much longer (see Fig. 2A). However, back-exchange of main-chain amides, which inevitably occurs during sample handling after quenching (i.e. peptic digestion and reverse-phase separation), interferes with HDX data analysis by diminishing the differences between exchanged populations and needs to be kept as low as practically possible. Recently, several attempts were made to alleviate the back-exchange problems during separation. In principle ultra performance liquid chromatography (UPLC) provides faster and superior resolution. Comparison between UPLC and conventional HPLC in HDX/MS experiments using cytochrome c as the benchmark protein failed to provide convincing evidence in favor of UPLC mainly due to higher back-exchange. This was caused by inadequate cooling of the injector in the commercial UPLC system [81]. A radically different approach using a supercritical fluid chromatography (SFC) that employs non-aqueous CO_2 _mobile phase and fast flow rates yielded substantial reduction of back-exchange compared to the HDX-optimized HPLC separation. The slightly impaired peptide separation in SFC was offset by the superior resolving power of the Fourier transform ion cyclotrone resonance mass spectrometer (FTICR-MS) which was used in this study [82].

#### Data analysis-from mass spectra to structural interpretations

**Figure 4:**
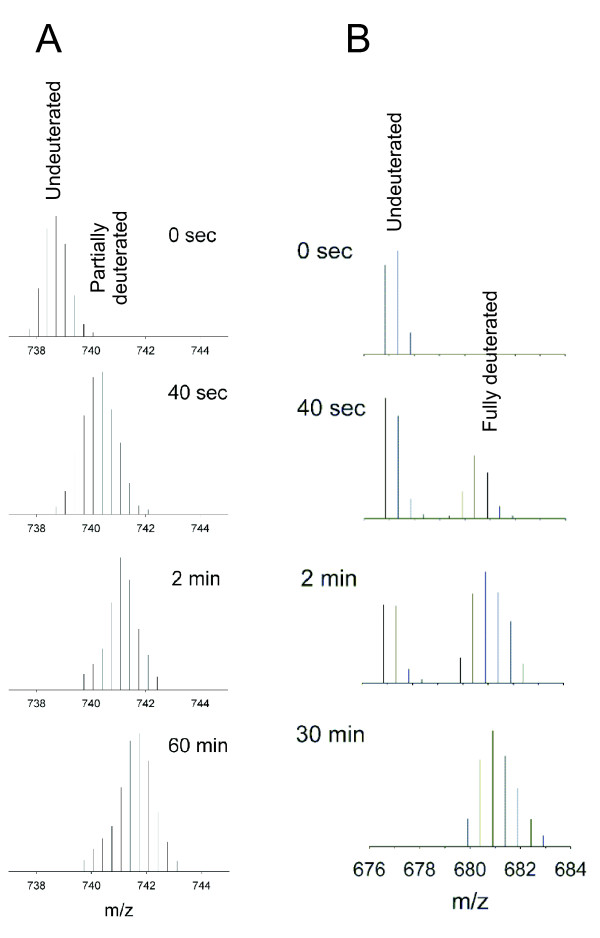
**Example of HDX detected by mass spectra.** m/z isotopic envelopes for EX2 (A) and EX1 (B) limit of exchange. Data obtained for a region that is situated within the subunit interface in ϕ8 P4 hexamer [114].

Digestion with pepsin or other acidic proteases is rather non-specific and under given conditions (e.g. digestion time, denaturant concentration, protease-to-protein ratio) yields overlapping fragments with lengths ranging from 5 to 15 amino acids. The same set of peptide fragments is obtained for a given protein and digestion conditions [75]. This assures that consistent and reproducible data sets are obtained in independent runs. Each of the fragments acquires one or more positive charges during ionization and produces a typical isotopic envelope in the mass spectrum (Fig 4A). The relative abundance of different ionization states and fragments strongly depends on the particular ionization interface and mass spectrometer design. Hence, it is advisable to use one MS instrument throughout the whole study.

Each peak in the envelope corresponds to an increasing number of heavier isotopes (naturally occurring ^13^C, ^34^S and the HDX introduced ^2^H) and spacing between them is inversely related to the acquired charge. Even the non-exchanged control (t = 0 s) contains series of peaks corresponding, in succession, to the monoisotopic fragment mass (the lowest m/z, all atoms are of the lightest isotope) followed by the peaks from species containing one or more of the heavier, naturally occurring isotopes. Note, that due to 1% abundance of ^13^C and other naturally occurring isotopes the monoisotopic peak is usually weak for longer fragments (>10 residues).

The fragments in non-deuterated sample are assigned to the amino acid sequence by tandem MS (MS^2^; i.e. fragmentation of the selected peptide in the mass spectrometer, usually performed by collision with gas or application of strong electric field, followed by analysis of the resulting ions; see [77] for details) [83]. In favorable cases, especially when using ultra-high resolution mass spectrometers (e.g. FT-ICR), assignments may be based on the exact mass alone. However, a confirmation by MS^2 ^is still desirable [84].

As expected, deuteration shifts the isotopic pattern to higher m/z values (Fig. 4A). As seen in the second spectrum from top the monoisotopic peak may not be resolved in the shifting isotopic envelopes and hence the patterns are usually identified by the expected progression of m/z peaks for the given charge state. One of the main advantages of MS detection is that MS spectra exhibit distinctly different patterns for EX1 and EX2 mechanisms. This provides basis for distinguishing between the two limits without measuring the tedious and often experimentally inaccessible pH dependence.

In the EX1 regime amides remain either all unexchanged or become all exchanged at once and thus the mass spectra show a bimodal pattern (c.f. t = 0 and 40 sec in Fig. 4B). The apparent rate constant, k_ex _= k1 in Eq. 2, corresponds to the rate of structural opening or a conformational change, and it is determined from the time-dependence of the integrated intensity belonging to the deuterated envelope.

In the EX2 regime exchange causes progressive shift and binomial broadening of the isotopic envelope (Fig. 4A). In this case the centroid of the isotopic envelope or the average of the peak positions weighted by their intensities are both reasonable and frequently used measures of the degree of deuteration [46,51,64,85,86]. In this case the HDX rate constant, k_ex _in Eq. 3, is obtained from the dependence of the centroid/average position on exchange time. This analysis provides kinetics averaged over the whole fragment and consequently corrections for the effects of primary structure (sequence) on k_2 _in the EX2 limit are impossible. Such correction is not necessary when comparing two states of the same protein, e.g. for mapping subunit interfaces in protein assemblies.

In certain cases (e.g. high resolution spectra like those shown in Fig. 4) the true deuteration distribution may be obtained from the measured envelope by deconvolution with the naturally occurring isotope distribution [87]. Such procedure allows analyzing exchange under conditions when part of the fragment is exchanging via EX1 mechanism while the rest belongs to an EX2 limit [88].

Site-specific resolution of exchange will be essential for quantitative study of enzymes and molecular motors and for direct comparison with theoretical results [89-91]. Several attempts to improve the resolution have been made. One approach was to analyze simultaneously the exchange kinetics of overlapping fragments and separate the individual contributions computationally. In favorable cases this may produce site-specific resolution for few residues within the entire sequence. An attempt at more reliable experimental solution was made by using MS^2 ^for sequencing of deuterated fragments. However, it was found that the collision-induced fragmentation step led to significant scrambling of deuterium among the amide sites and hence this approach proved impractical [74]. Recent experiments using gentler fragmentation methods demonstrated significant preservation of the deuteration pattern [73,92].

In all cases the measured degree of exchange is always lower than the actual value because some of the label is lost during sample handling (e.g. digestion and HPLC) due to the back-exchange. The true extent of exchange is an important parameter for interpretation. Take the following example: In the absence of site-specific resolution the exchange is averaged over the whole peptide fragment. Hence, situations, in which half of the fragment residues are exposed and exhibits fast exchange while the rest is buried and does not exchange at all, are common. Ignoring the true extent of the exchange, which amounts to only half of the available sites in this example, and taking into account only the fast kinetics would lead to an erroneous conclusion that the whole peptide is exposed.

In order to obtain the true extent of exchange one must correct for the back-exchange. The degree of back-exchange depends on the experimental setup and sample handling and varies from as little as 10% for ESI-MS to about 40% in MALDI-TOF MS detection. There is also variation between different fragments, presumably due to sequence effects on the back-exchange. Therefore, the best way to correct for back-exchange is to measure a fully deuterated control sample under identical conditions, e.g. a protein which was first denatured and then re-folded in D_2_O buffer. For some proteins refolding in D_2_O is impossible and one must resort to approximations. For example, it is fair to qualitatively compare the relative extent of exchange for two states of the same protein (e.g. assembled and free subunit of a virus) if the two data sets were collected under identical conditions [70].

#### Automated data processing

Mass spectrometry is venerable for generating large data sets and HDX-MS is no exception. A typical 35 kD protein may yield about 100 assigned fragments which contribute to the mass spectra. In addition, the spectra contain plethora of unassigned peaks e.g. from oxidized protein or from pepsin. In HDX-MS the useful MS data is spread over several (4–10) scans within the LC-MS run. Usually, the full kinetics contains at least 15 time-points collected in three independent replicas. Taken together one needs to extract and process at least 4000 isotopic envelopes from about 200 spectra per each kinetic curve. Such a task calls for a considerable automation and deployment of data-mining tools.

MS^2 ^peptide assignments can be done using either commercial proteomics add-on packages for automated database searches and subsequently validated by spectral prediction tools when necessary [93]. Despite the apparent popularity of HDX-MS, specific tools are not to be found in any of the commercial software packages that are currently supplied with instruments. This spurred in-house development of various HDX-MS-specific software packages [54,79,94] and eventually led to applications ranging from semi-automated to fully-automated processing that are now freely available [88,95,96]. The utility of some packages is still hindered by the plethora of proprietary data formats used by different manufacturers but could be overcome by adopting the mzXML standard for data interfacing [96,97]. Recently, Pascal and colleagues launched a web application ("Deuterator") compatible with multiple file formats for automated HDX-MS data analysis [96,98]. Although promising, the utility of the web-based approach is somewhat compromised by the need to supply large datasets to a remote server.

## Applications

In the following sections we present selected examples of HDX-MS use in detection and characterization of folding intermediates, association of small oligomers and dynamics in large assemblies. Later we provide illustrations of HDX-MS utility for structural characterization of aggregates and intrinsically unstructured proteins. Finally, we discuss applications to characterize functional dynamics in protein complexes and molecular machines.

### Detection of folding and association intermediates in vitro

#### Native state exchange reveals folding intermediates

Multi-state and two-state protein folding cannot be often distinguished due to instability of partially folded intermediates. Meta-stable intermediates can be detected via HDX and play a crucial role in the determination of folding kinetic and native state dynamics [85]. HDX experiments are also considered most effective in teasing out the structural details of protein folding intermediates, often with amino-acid resolution [99].

The three-state unfolding of ubiquitin (highly structured, partially unstructured A-state, and fully unstructured state) was examined by HDX-MS [100]. The highly dynamic A-state consisted of flexible, rapidly exchanging C-terminal region while the N-terminal domain adopted less dynamic, native-like β-strand configuration.

#### Time-resolved techniques and characterization of folding and assembly intermediates

**Figure 5:**
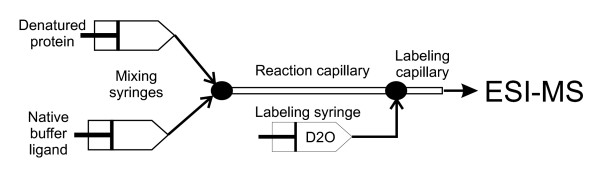
Schematics of on-line folding and pulse labeling apparatus (based on [102]).

Pulse-labeling HDX-MS techniques enable detection and characterization of transient folding intermediates which are not readily populated under equilibrium conditions [101]. An on-line HDX pulse-labeling ESI MS apparatus was developed and tested on myoglobin folding and heme incorporation [86,102] (Fig. 5). The method is based on tandem mixing chambers: the first one serves to initiate folding/assembly reaction while the second one is used to stop the folding reaction and transiently expose the products to deuterium label. Subsequently, the exchange is quenched and the incorporated label is quantified by MS. This provides a "snapshot" of protection at a particular stage of the reaction. Usually, this is applied to intact proteins but there is no principal obstacle in carrying out pepsin digestion and LC-MS analysis and obtain region-specific exchange kinetics.

The method enabled millisecond resolution of the folding dynamics for a well-studied model protein ubiquitin and led to the discovery of a previously uncharacterized intermediate [103]. The method was then used to follow folding and association of dimeric protein, S100A11, and revealed three different kinetic species – a relatively unfolded monomer, a more compact folded monomer and the native dimer [104].

Pulse labeling can also reveal the interplay between folding and assembly in multidomain, multimeric proteins. Rabbit muscle aldolase is a homotetramer encompassing subunits with three domains each. A locally unfolded aldolase was pulse-labeled with deuterium after destabilization in urea for a defined period of time (1 min to 48 h). Isotope patterns revealed four populations corresponding to distinct conformations: one native and three distinct, partially unfolded intermediates. The intermediates, which were further characterized using pepsin digestion, corresponded to aldolase with one, two or all three domains unfolded, respectively. Remarkably, the completely unprotected intermediate, which also lacked secondary structure, still remained tetrameric, suggesting strong coupling between folding and assembly of the tetramer. The observed HDX kinetics supported sequential and cooperative mechanism of aldolase unfolding [51,80,105].

#### Structural characterization of expressed proteins

**Figure 6:**
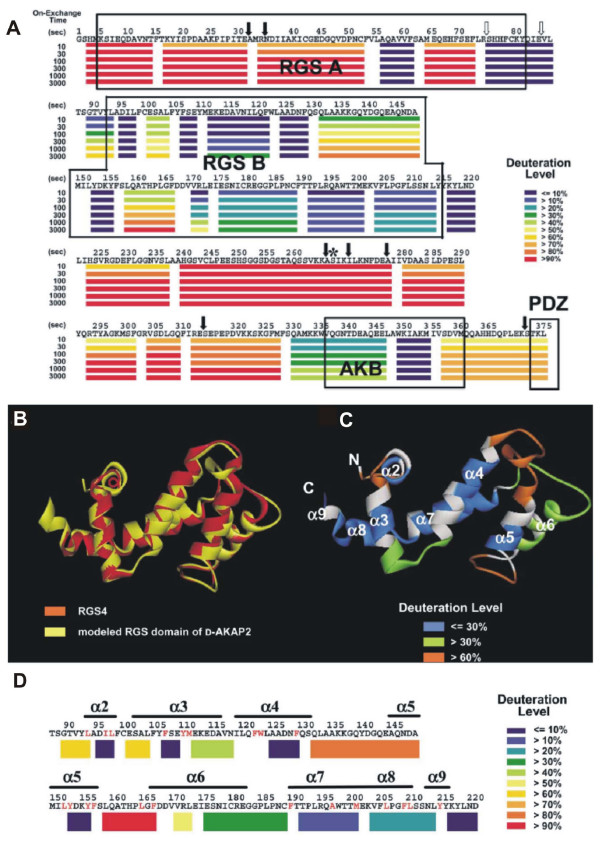
**Domain structure of D-AKAP2 scaffolding protein.** (A) Graphical representation of deuteration levels at different time points is shown in color (scale on the right) below the sequence. (B) Backbone homology-based model of the RGS domain (C) Color representation of deuteration levels after 3000 s mapped onto the modeled structure. (D) Deuteration levels after 3000 s mapped onto the primary structure. From [106] with permission.

When there is no high resolution structure available, HDX-MS experiment can provide valuable information about the domain structure and may be used to validate structural models which are built solely on the basis of limited sequence homology. This approach was applied to a protein kinase scaffolding protein, D-AKAP2, which augments interactions of signaling molecules inside cells [106]. HDX-MS revealed two regions of low exchange located in the midst of fast exchanging regions and identified two distinctly folded regions (Fig. 6). Fig. 6A illustrates a plausible representation of region-specific HDX kinetics in the absence of three-dimensional structure. The sequence, which was found homologous to regulator of G-protein signaling (RGS), mapped within the first folded region. The second folded region encompassed a highly protected protein kinase A (PKA) binding site and a less protected PDZ-binding motif (PDZ domain is a potential target of D-AKAP2). HDX-MS thus confirmed the multi-domain architecture of D-AKAP2. HDX-MS was also used to validate a homology-based structural model of the RGS domain (Fig. 6B–C) [106].

An interesting application of HDX-MS has been developed to follow protein folding during in vitro, cell-free, transcription-translation [107]. This is a fast and easy way to use method based on MALDI-TOF and yields global degree of folding using minute protein quantities without purification. In addition, it affords rapid identification of the expressed protein. HDX-MS was also used to refine protein constructs for crystallization trials [108,109]. A somewhat similar MALDI-MS approach was used to characterize stability of an expressed protein in the cell extracts i.e. under conditions close to those found in the cytoplasm [38]. The method, abbreviated by the authors as SUPREX (stability of unpurified proteins from rates of H/D exchange) revealed that the model protein, bacteriophage λ repressor, exhibited identical stability in the cell lysate and in the dilute solution of pure protein, respectively. This study was later extended to perform and detect HDX of the λ repressor in the cytoplasm of intact *E. coli *cells, i.e. *in vivo *[37]. This method exploited permeability of lipid membranes for small molecules like water and urea which allowed for rapid equilibration of D_2_O between the cytoplasm and the deuterated medium containing increasing amounts of denaturant. As in their previous study the authors found no difference between repressor stability under *in vivo *and *in vitro *conditions, respectively. However, the *in vivo *stability was significantly enhanced by administering hyperosmotic shock to the cells prior to the exchange experiment.

### Assembly and dynamics of large complexes

Macromolecular complexes, some of which are indeed fairly sophisticated molecular machines, ensure consecutiveness of cellular processes such as macromolecular synthesis, transport, and metabolism. Their functions rely on self-assembly, subunit rearrangements and conformational changes throughout the duty cycle. Viruses represent special class of such machines and, in effect they could be considered smart containers for targeted delivery of macromolecular cargo. They are programmed for controlled replication, encapsidation, transport and release of their genomes into new host cells. This is accomplished by series of concerted structural changes within viral capsids. The utility of HDX-MS in virus research and in characterization of macromolecular complexes in general was recently reviewed [71] and here we present selected examples, mostly from the virus field, to illustrate the type of problems this method may help to answer.

#### Mapping subunit interfaces and association dynamics

**Figure 7:**
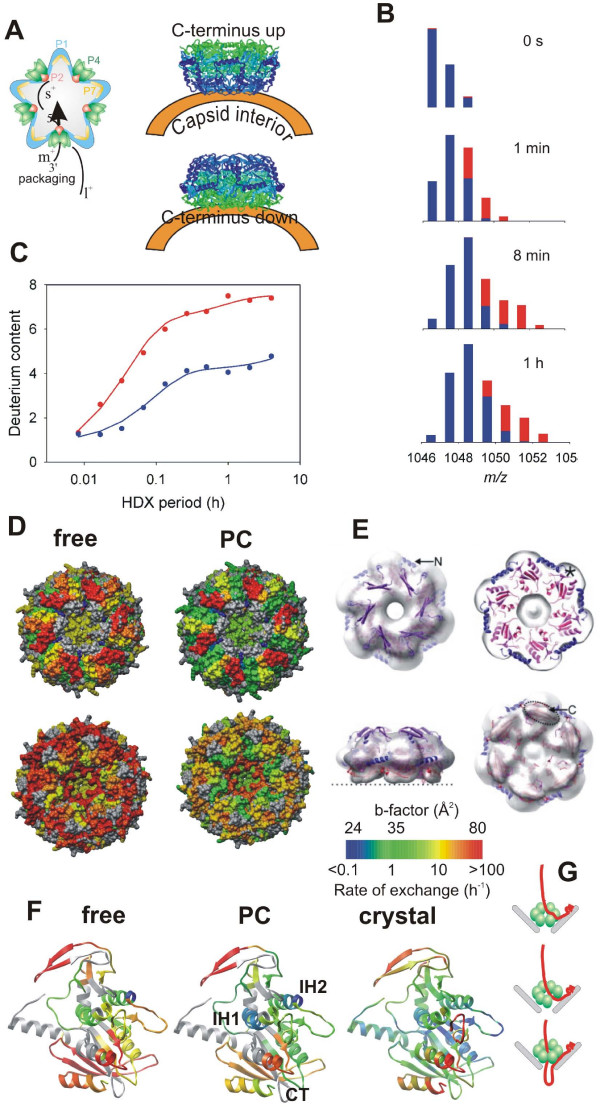
**Mapping subunit interfaces and dynamics in large viral assembly- P4 hexamer within ϕ12 procapsid [94].** (A) Schematics of the viral procapsid (PC) and packaging of ssRNA precursors by the P4 hexamers associated with the capsid vertices (left). The right panel illustrates the problem of determining the orientation of the hexamer with respect to the procapsid. (B) Bar representation of the isotopic envelopes during progressive deuteration, free hexamer in red and PC-bound in blue. (C) The HDX kinetics derived from centroid positions of the isotopic envelopes in panel B (and from additional data not shown). (D) Mapping of average exchange rates onto P4 structure in surface color representation (color scale on the bottom right). (E) Fitting of the P4 hexamer into the EM-derived electron density using the correct orientation from HDX experiment (adopted from [113]). (F) Average exchange rates mapped onto the ribbon diagram of P4 subunit for free (left) and PC-bound (middle) hexamer together with the scaled crystallographic temperature factors (B-factors, right panel). Color scale as in panel D. (G) Schematics of ssRNA loading into the hexameric packaging motor via ring opening mechanism. Top: A specific viral RNA structure (packaging signal) is recognized by the major capsid protein P1 which brings the polynucleotide strand to the vicinity of P4. Middle: P4 ring opens and lets the RNA slip in between subunit interfaces into the central channel. Bottom: The ring topologically encloses the bound RNA and translocates RNA in 5' to 3' direction into the capsid at the expense of ATP hydrolysis (packaging).

The first step towards functional macromolecular complex is assembly of subunits guided by subunit-subunit interactions. In many instances only the high-resolution structures of subunits are available while a medium resolution electron density of the whole assembly is readily obtained by cryo-EM. The fitting of the subunit structures into the EM density is greatly facilitated by knowing the subunit interfaces within the complex. This strategy was adopted for bacteriophage ϕ12 which is a dsRNA virus belonging to the *Cystoviridae *family and is structurally related to members of the *Reoviridae *family [110]. Although the high-resolution structure of the virus-associated packaging ATPase, a hexameric molecular motor P4, had been known [111] and the hexameric structure had been resolved by cryo-EM [112,113] it was not possible to determine which way the hexamer interacted with the viral procapsid (Fig. 7A). HDX-MS was used to map the subunit interfaces [94]. Fig. 7B shows comparison of the isotopic patterns for the C-terminal helix in the free hexamer (red) and the PC-bound motor (blue). A substantial increase in protection (lower final plateau in Fig. 7C) and slower kinetic (smaller initial slope) is clearly apparent for the assembled state. Mapping of the average exchange rates for all resolved fragments onto the surface representation of the hexamer confirmed that P4 associates with the procapsid using its C-terminal facet (Fig. 7D). This information was subsequently used to fit the atomic model of the hexamer into a refined asymmetric cryo-EM reconstruction of a related virus ϕ6 [113] (Fig. 7E).

HDX-MS also revealed that the association of the motor with the viral capsid stabilized subunit interfaces (c.f. exchange of helices IH1 and IH2 in Fig. 7F) and prevented spontaneous opening of the hexameric ring (Fig. 7G). Ring opening had been detected by HDX-MS and associated with loading of ssRNA into the central channel (Fig. 7G) [114]. The exchange dynamics of the PC-associated hexamer closely followed temperature B-factors derived from the crystal structure while the free, solution state was more dynamic [94] (Fig. 7F).

HDX-MS and crosslinking was used to identify new contacts between subunits in the capsid of human immunodeficiency virus (HIV) [70]. The subunit of HIV capsid (CA) encompasses N-terminal domain connected via a linker to C-terminal domain. The capsid assembly process was thought to be driven by association (dimerization) of C-terminal domains and hexamer formation by six N-terminal domains. While the C-terminal domain was known to form stable dimers in solution there was no evidence for the oligomerization of the N-terminal domain. Hence, the assembly mechanism was far from being certain. By comparing the exchange of the full-length CA in the monomeric and the assembled state Lanman and colleagues were able to delineate a new interface between the N-terminal and C-terminal domains of neighboring subunits [70,115]. This contact was shown to play essential role in stabilizing hexamers during assembly.

HDX-MS can also provide information about subunit association kinetics in functional assemblies. Small heat shock proteins (sHSP) belong to a family of molecular chaperones, which transiently bind partially unfolded proteins and prevent their aggregation. One member of this family, HSP16.9, assembles into a dodecamer at room temperature. Surprisingly, subunit interfaces exhibited no protection against HDX after incubation for 5 s in D_2_O [50]. Note, that under these conditions most oligomeric proteins show significant protection of their subunit interfaces. Almost complete exchange suggested that large conformational motions were taking place within the assembly, leading to the disruption of subunit interfaces. When pulse-labeled for just 10 ms, the subunit interfaces showed significantly lower exchange comparing to the 5 s experiment. Thus, the HSP16.9 oligomer underwent fast association-dissociation dynamics on a sub-second time scale. At 42°C, HSP16.9 forms a dimer with the same exchange pattern and kinetics as the dodecamer at room temperature. It is believed that HSP16.9 is heat-activated by shifting the equilibrium between the two forms [50] and the fast association-dissociation dynamics plays essential role in the process. This example also shows that it is important to consider HDX kinetics on millisecond time scale when dealing with metastable, highly dynamic complexes.

#### Structure and dynamics of subunits within large assemblies

EM and X-ray diffraction yield structures of well-defined, stable conformations. However, even highly symmetric, icosahedral viral capsids are dynamic entities. Essential biological processes, like delivery of viral genomes, are mediated by structural transformations. HDX-MS enables monitoring of these dynamic events.

**Figure 8:**
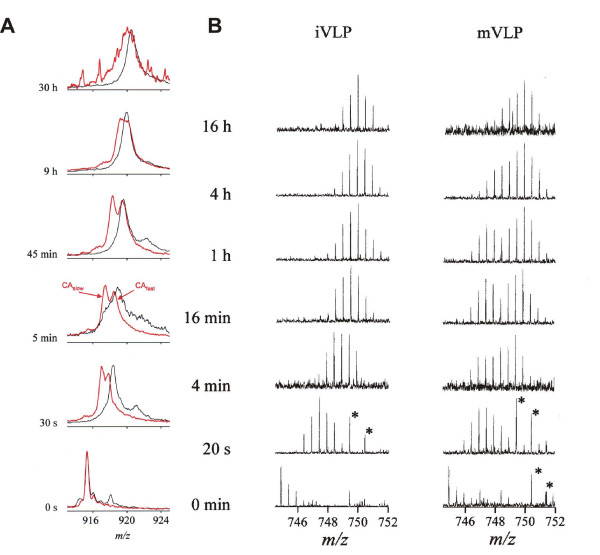
**Structural changes during HIV maturation [71, 115, 116]. **(A) Progress of deuteration for the intact capsid protein (+28 charge state, 25 601 Da) in mature virus-like-particles (mVLP, red) and as disassembled monomer in solution (black). (B) Time-resolved isotopic envelopes for the peptic fragment encompassing residues 55–68 of the CA protein in immature (left) and mature (right) VLPs. The peaks marked by asterisk do not belong to the CA fragment envelopes. From [71] with permission.

Many viruses require a maturation step in which a capsid precursor undergoes large scale structural rearrangements in response to e.g. protease processing of the viral polyprotein. HIV maturation is triggered by a virion-associated protease which specifically cleaves the assembled Gag polyprotein to releases the individual structural proteins (NC, nucleocapsid; CA, capsid; MA matrix; ENV, envelope glycoprotein). During the process the assembled CA collapses from a spherical form into a conical core. This step is essential for infectivity and hence the protease has been successfully targeted by several antiviral drugs. However, in structural terms, the maturation process has been elusive [115]. HDX-MS was used to compare CA in the mature and immature form of virus-like particles (VLP). The intact CA protein in the mature form exhibited significant protection with respect to the immature state (Fig. 8A) [116]. A bimodal pattern in the m/z spectra of the intact, mature CA indicated two populations of CA conformers. The first exhibited protection similar to the immature CA while the second was significantly more protected. Similar bimodal HDX pattern was observed for a peptide fragment from the interface between N- and C-terminal domain (Fig. 8B). This demonstrated that only half of the subunits in HIV-1 capsid matured into the conical cores and that the formation of the heterotypic N-C interface is a key feature of the maturation process. On the other hand, the formation of new interfaces between N-terminal domains, which had been proposed on the basis of EM to mediate maturation, was not detected [116].

Unlike HIV dsDNA and dsRNA bacteriophages (e.g. *Cystoviridae *discussed above) mature via packaging of their genomes into empty procapsids which simultaneously expand and undergo substantial stabilization [117,118]. HDX-MS application to viral maturation was pioneered using dsDNA bacteriophages P22 and Raman spectroscopy for detection [29]. This initial investigation was subsequently extended by mass spectrometry [33] and demonstrated that the observed stabilization of the viral lattice was due to refolding of coat protein domains. Further study of P22 capsids by FT-ICR HDX-MS identified the N-terminal domain as being substantially stabilized upon maturation while the C-terminal dimerization domain remained unaffected. Together with crosslinking experiments HDX results assisted mapping of the refolded domains into the EM-derived electron density [119,120].

Flexibility of virions has been demonstrated as an important factor in the delivery of genomes by plus-sense ssRNA viruses [121]. Capsid dynamics of human rhinovirus was studied by HDX-MS [122]. A fast and intermediate exchange pattern was observed for the interfaces around the five-fold axis. These sites may serve as the initiation site for uncoating and release of the viral RNA.

Native-state HDX of whole protein subunits within the intact large and small ribosomal subunit was detected by MALDI-MS [66]. While this method did not resolve subunit interfaces it provided first glimpse at the overall dynamics for almost all ribosomal proteins. The protection correlated well with the assembly pathway i.e. subunits that were incorporated early during ribosomal assembly exhibited significantly higher protection. On the other hand, faster HDX was observed for subunits implicated in tRNA translocation between sites or those involved in pivoting of large and small ribosomal subunits. These functions presumably require higher flexibility.

### Aggregation, amyloid and inclusion bodies

Fluids in cells contain macromolecules at high total concentration, causing molecular crowding [123]. In most cases crowding favors protein folding as well as formation of functional complexes relative to the less compact, non-native structures. However, under such conditions even brief loss of native protein structure or exposure to extreme conditions (e.g. oxidative stress, heat shock) may lead to aggregation. Aggregation produces plethora of species, ranging from soluble oligomers, amorphous aggregates, to fibrils, amyloid plagues and inclusion bodies [124,125]. HDX-MS has played important role in characterizing structure and dynamics of protein aggregates which are often refractory to other methods [126]. HDX-MS also constitutes an indispensable tool in characterizing intrinsically unfolded proteins [127].

Prion proteins are prone to adopt different structures and some mutants readily oligomerise and aggregate to form amyloid plagues. Amyloid and inclusion body formation is associated with severe human disorders such as Alzheimer's or Huntington's diseases. The oligomers are currently thought to constitute the toxic species.

One of the proteins that are often associated with amyloid formation is α-synuclein. HDX-MS was used to delineate structural differences between two states of α-synuclein, the natively unstructured soluble monomer and the aggregated insoluble amyloid [128]. The monomer exchanged with rates corresponding to an unstructured random coil. In the amyloid state the long N-terminal and the C-terminal segments remained mostly unprotected while the central β-sheet was significantly protected. The protected β-sheet segments exceeded the length expected for an amyloid ribbon and no exposed amides corresponding to the putative interconnecting turns were observed. These results indicate that the α-synuclein amyloid adopts a structure similar to that of amyloid-β [129].

Ovine prion protein oligomerization was investigated by a combination of size-exclusion chromatography, circular dichroism and HDX-MS [130]. Three different oligomeric species were detected and structurally characterized. Surprisingly, HDX detected increased flexibility of certain regions that were shown to play essential role in aggregation. The heterogeneity and the increased dynamic character of these oligomeric precursors pointed to the existence of multiple aggregation pathways.

Overexpression and inclusion body formation may facilitate high recovery of bioactive protein provided the protein attains native structure after solubilization [131]. Over-expression and purification from inclusion bodies was employed to study the cytoplasmic region of tyrosine kinase-interacting protein (TIP) from Herpes virus saimiri. Intrinsic disorder was predicted for TIP and confirmed by HDX-MS but this did not compromise the enzyme activity and binding [132].

### Protein dynamics and function

Protein function is often intimately linked to protein dynamics and involves, for example, conformational changes during enzyme activation or segment immobilization imposed by ligand binding. Conformational changes require transient population of higher energy states on the folding landscape and thus they can be detected by HDX as in the case of folding intermediates [133-135]. The importance of thermally activated protein dynamics was demonstrated for a thermophilic alcohol dehydrogenase by measuring temperature-dependent HDX rates [136]. The activation energies for HDX rates of peptides in the vicinity of the NAD^+ ^cofactor and the substrate binding site underwent two transitions, at 30 and 45°C, respectively. These transitions correlated with changes in NAD^+ ^binding kinetics and enzymatic activity.

**Figure 9:**
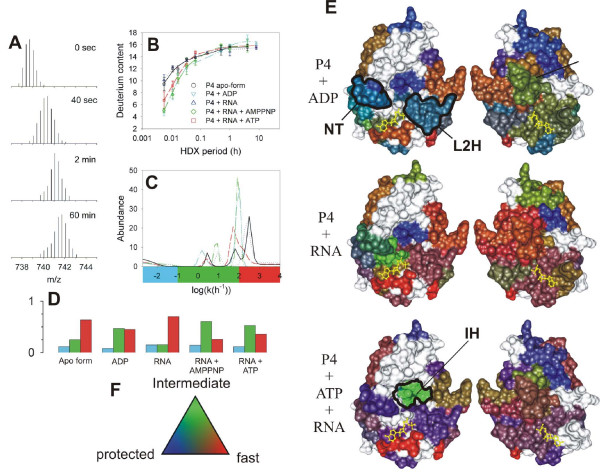
**Mapping functional dynamics in the hexamer of P4 from bacteriophage ϕ8 [114].** Mass/charge (*m/z*) spectra corresponding to the peptic fragment encompassing residues 139–158 (*m *= 2210.14 Da, *z *= 3) during H/D exchange (only interval 0 to 60 min shown). (B) Increase of deuterium content in the peptide (symbols) and the corresponding maximum entropy fit (MEM) for P4 alone (black circles, solid line), P4+1 mM poly(C) (blue triangles, dotted), P4 + 1 mM poly(C) + 1 mM ATP (red squares, dashed line), P4 + 1 mM poly(C) + 1 mM AMP-PNP (green diamonds, dash-dot-dot) and P4 + 1 mM ADP (cyan inverted triangles, dash-dot). Standard deviations (error bars) were estimated from three independent experiments. (C) Rate distributions obtained by MEM fitting of data in panel B. The color bar under the panel indicates the three integration regions which were used to obtain the number of sites within each rate class (blue = slow/protected, green = intermediate, red = fast/unprotected). (D) Number of amide sites in the three classes and under different conditions (nucleotide di/triphosphates, RNA binding) obtained from data in panel C, bar colors as in panel C. (E) RGB representation of the HDX kinetics for subunit interfaces. The two facets (left and right) represent the facing interfaces from the neighboring subunits in a surface representation. Bound ATP molecule is shown in yellow ball-and-stick representation. Several regions of interest are delineated: NT-nucleotide binding pocket; L2H-loop 2 and α-helix 6 which constitute the moving lever of the motor; IH-interfacial helix which becomes transiently exposed during ring opening and RNA loading. (F) Three-color, RGB scale for number of amides exchanging in the three classes.

HDX-MS was used for mapping the interactions of proteins with ligands [137,138] and enabled to unravel subtle structural changes in troponin C upon Ca^2+ ^binding. These changes are important for regulation but escaped detection by other techniques [139]. The concept is illustrated in Fig. 9A–D for binding of nucleotide di- and tri-phosphates and RNA to the hexameric viral packaging motor, protein P4 (see above). The exchange of a peptide fragment originating from the vicinity of the ATP binding site exhibited slower rate in the presence of ATP or ADP but is was largely unaffected by RNA binding (Fig. 9B–D) [114]. Further insight was obtained by computing the distribution of exchange rates by a maximum entropy method [140] (Fig. 9C) and by classification of the amide sites into three groups: fast (exchanged before the first time point, i.e. 30 sec), intermediate (exchanging on the time scale of the experiment), and protected (not exchanged during the whole duration of the experiment, i.e. 8 h) (Fig. 9D) [114]. The three-class analysis revealed that reversible ligand binding affected mostly the fast exchanging amides and provided only partial protection. This is understandable given that ATP and ADP binding and dissociation both happen on millisecond-to-second time scale.

The three-class analysis proved valuable in visualizing changes in local stability during RNA binding and ATP-driven translocation by the hexameric packaging motor P4. HDX information for each peptic fragment was color-coded and mapped onto the three-dimensional structure. For each fragment the applied color was an RGB (red-green-blue) blend which was weighted by the relative fraction of protected (blue), intermediate (green) and fast (red) amide sites (Fig. 9F). Such representation is superior to the simple average rate coloring scheme used in Fig. 7 because it contains more information and enables to distinguish cases in which the average rate is not affected, e.g. when fast and slow sites shift to intermediate class. The three-color representation revealed increased flexibility of the hexamer upon RNA binding (Fig. 9E). During translocation the protein subunits cycle between stable and flexible states and this resulted in purple color of many regions (purple = blue + red). The only exception is the interface helix (IH, green) for which the intermediate exchange is of the EX1 type (see the primary MS data in Fig. 4) and represents opening of the ring during RNA loading.

HDX-MS proved extremely useful in uncovering allosteric activation networks in protein kinases [141,142]. The extracellular regulated protein kinase-2 (ERK-2) is a MAP kinase which is activated by phosphorylation. The influence of phosphorylation on conformational flexibility was probed by HDX-MS [143]. HDX demonstrated that phosphorylation induced flexibility in the hinge region between two domains. Subsequently, conformational changes upon AMP-PNP binding to the inactive and the activated (phosphorylated) ERK-2 revealed that the DFG motif within the catalytic site was stabilized in the presence of AMP-PNP but only in the active form [144]. Hence phosphorylation facilitates interdomain closure that is necessary for the precise alignment between ATP and the polypeptide substrate.

In another example HDX-MS was used to probe allosteric activation of coagulation factor VIIa (FVIIa) by a tissue factor (TF), both of which play essential role in blood clotting [145]. Observed HDX kinetics demonstrated stabilization of the activation domain and the 170-loop in FVIIa upon TF binding. Interestingly, the two protected regions are distal to the TF recognition helix within FVIIa sequence. Comparison of HDX results with molecular dynamics simulations identified a key interaction between Leu305 and Phe374 which is likely to transmit the stabilizing effect from the recognition site to the activation domain. This is an example in which region specific HDX was augmented by molecular dynamics simulations to reach conclusions at atomic level.

## Conclusion

It is clear from the above illustrative but largely incomplete survey of HDX-MS applications that this method has gained popularity within the community. As suitable mass spectrometers become more affordable they will be acquired by individual labs for dedicated HDX-MS use. This will generate vast amounts of data and hopefully stimulate further software developments. However, at the moment there is no public depository for such a vast amount of potentially useful data. The results are scattered throughout literature in various formats which are generally not amenable to quantitative comparisons and searches. Therefore, there is an urgent need to develop a uniform way of how to present and deposit HDX-MS results and time is ripe to create a fully searchable public database.

In addition, more advanced instrumentation will enable developments of new methods and improve sequence coverage and site-specific resolution by e.g. tandem MS. Another avenue is to improve the overall throughput by decreasing the time necessary for data collection by e.g. lab-on-chip implementation of fully automated protocols. This in turn would significantly enhance temporal resolution of HDX and enable probing faster, functional dynamics which is often associated with enzyme action. These later developments will make HDX-MS valuable companion to structural genomics.

## Authors' contributions

BS selected the reviewed papers and drafted the manuscript. RT wrote the introduction and edited the final version. All authors read and approved the final version.
